# Exogenously applied 5-aminolevulinic acid modulates growth, yield, and physiological parameters in lentil (*Lens culinaris* Medik.) under rain-fed and supplemental irrigation conditions

**DOI:** 10.1038/s41598-023-48732-y

**Published:** 2023-12-03

**Authors:** Hamid Mohammadi, Parviz Ezati, Saeid Hazrati, Mansour Ghorbanpour

**Affiliations:** 1 Faculty of Agriculture, Azarbaijan Shahid Madani University, Tabriz, Iran; 2 Department of Medicinal Plants, Faculty of Agriculture and Natural Resources, Arak University, Arak, Iran

**Keywords:** Physiology, Plant sciences

## Abstract

Lentils are a significant source of plant protein and are cultivated across Asia, Europe, and North Africa. Plants are subjected to various environmental stresses, which can hinder growth, yield, and productivity. 5-aminolevulinic acid (ALA) is a compound that acts as a precursor in the biosynthesis of tetrapyrroles and can increase plant tolerance to different abiotic stressors. However, the effects of exogenously applied ALA on lentil growth, yield, and physiological parameters under rain-fed and supplemental irrigation conditions are not well-known. In this study, a split plot experiment was conducted to investigate the impact of ALA foliar application and supplemental irrigation on lentil (*Lens culinaris* Medik.). The experiment was designed based on a randomized complete block with three replications. The main plot included four levels of supplemental irrigation [(supplementary irrigation in the flowering and early seed-filling stages, supplementary irrigation in the flowering stage, supplementary irrigation in the early seed-filling along with rain-fed conditions (no irrigation)]. The subplot considered foliar application of ALA at varying levels [(0 (control), 50 and 100 ppm)]. The results showed that water regimes and foliar spray with ALA significantly (*P* ˂ 0.01) affected plant height, number of pods per plant, pod weight, number of seeds per pod and weight of 1000 seeds, biological yield, seed yield, and harvest index. The highest total chlorophyll content was observed in plants that were subjected to supplementary irrigation in flowering and early seed filling stages and foliar sprayed with 100 ppm ALA. The study also found that exogenous ALA improved drought tolerance in lentil plants under rain-fed conditions mainly by regulating antioxidant enzymes, which ultimately protected the cellular membranes against overproduction of H_2_O_2_. Furthermore, ALA application increased total carbohydrate contents at all supplemental irrigation levels, but the rate was higher in complementary irrigation conditions during flowering and early seed-filling stages. Malondialdehyde (MDA), H_2_O_2_, and proline contents were increased in field-grown plants under rain-fed conditions without exogenous ALA application. In conclusion, this study sheds light on the effects of ALA foliar spray and supplemental irrigation on lentil growth, yield, and physiological parameters. The findings suggest that exogenous ALA can improve plant tolerance to various abiotic stressors and enhance plant growth, yield, and physiological parameters.

## Introduction

Drought has a strong impact on agricultural production worldwide, especially in areas with low rainfall. The way plants react to water scarcity depends on various factors, such as the frequency of dry and wet periods, soil and atmospheric water deficits, and the severity and timing of drought events^1^. When drought occurs, crop production and yield potential decrease significantly. By 2090, it is estimated that 30% of the earth's land will be exposed to severe droughts. A report by the World Bank states that drought is the most expensive natural disaster, causing economic losses every year to communities that rely on agriculture. Rain-fed agriculture, which accounts for 80% of the world's cultivated land, is particularly vulnerable to drought. It is responsible for about 60% of global crop production, according to the FAO report^2^.

The reduction in food grain production could result in food shortages and a decrease in nutrition security. The major reasons for declining food productivity trends are the limitations of water resources and available water. Rain-fed farming crops have low yields due to not considering the principles of dry farming, and the lack and inappropriate distribution of rainfall. Enhancing rain-fed farming techniques to optimize production and water management leads to the improvement of soil, water, and product quality^3^. To solve this problem, or at least reduce its effects, a special strategy should be taken. In the first step, an important strategy could be replacing plants with high-water demand with plants that require less water but have high water-use efficiency. Therefore, it is necessary to investigate the reasons for the greater resistance of specific plants to stress factors and to determine the mechanisms involved in improving plant tolerance. In the next step, creative solutions should be sought to address the weaknesses of plants and increase their production and performance under such conditions^4^. One of the methods and effective strategies for agricultural practices in reducing risk and increasing production, as well as improving the degree of stability and sustainability in rain-fed farming, is supplementary irrigation management^5^.

Legumes are an excellent source of protein, carbohydrates, minerals, and dietary fiber, and are considered the second most important source of human food after cereals. These plants can prevent soil erosion by fixing nitrogen biologically, while also improving soil fertility. They are used as cover crops or in rotation with many other agricultural plants, and play a vital role in the stability of agricultural systems. With the growing global population and increasing food demand, there is a need to increase the production of plant-based protein materials, which are valuable sources of nutrition. Therefore, the production of legumes has received special attention as a healthy source of protein and vitamins^6^. However, the agricultural production system is facing several adverse climatic and biogenic conditions.

Lentil (*Lens culinaris* Medik.) is a type of legume that thrives in cold environments and is a long-day plant that grows in temperate climates. It is a popular human food and is cultivated in various regions worldwide, from plain areas to high altitudes of up to 3500 m. Lentil seeds are rich in proteins, minerals, vitamins, and other essential food elements. Despite its many benefits, lentil production faces numerous environmental challenges, with water deficit stress being the primary issue that decreases its yield globally^7^. Since most grain legumes, such as lentil, pea, vetch, and bean, are typically grown in rain-fed farming systems, they are more susceptible to drought conditions^8^.

There are several methods used for developing stress-tolerant plants and improving yield. These include breeding, genetic engineering, and the use of plant growth regulators (PGRs). Lately, exogenous application has been given more attention. One of the PGRs that has aroused interest is 5-aminolevulinic acid (ALA). ALA is a highly promising chemical compound that has been found in various organisms, including bacteria, algae, plants, and animals. It acts as a precursor to essential compounds such as vitamin B12, chlorophyll, heme, and phytochrome in plants. ALA plays a significant role in plant growth and development, enhancing their photosynthetic capacity even under adverse conditions^9^. Studies show that exogenous application of ALA regulates the activity of enzymatic and non-enzymatic antioxidants, enhances the plant's resistance to stress in dry farming conditions, and reduces the adverse effects of stress^10^. Additionally, ALA treatment delays the senescence of *Pseudostellaria heterophylla* and enhances the photosynthetic capacity of tea trees under drought stress^11^. It has been showed that ALA treatment could significantly increase the pigment content, alleviate PSII photodamage, and enhance the photosynthetic capacity of tea trees under drought stress^12^. However, there is no report of the use of ALA in rain-fed conditions, especially in legume production systems. Furthermore, the appropriate concentration and mode of application of ALA in agricultural systems are still areas that require exploration.

Several factors contribute to the low yield of lentil plants in rain-fed conditions. Among them, the amount of annual precipitation and the supply of moisture in the sensitive stages of plant growth are essential. The lack of moisture can affect numerous aspects of plant growth and development, such as cell turgor, stomatal opening and closing, photosynthesis, respiration, and transpiration, as well as processes that depend on the presence of water like enzyme activities. Many reports suggest that the disruption of various physiological processes, reduction of plant hormone synthesis, carbon and nitrogen metabolism, and changes in the structure and activity of proteins occur due to the lack of water. The limitation of photosynthesis is the primary cause of the reduction in crop growth under drought stress conditions^13^. Considering the adverse effects of drought and lack of water supply, supplementary irrigation can be a suitable approach to improve the growth and yield of dryland crops. Moreover, introducing plant growth regulators (PGRs) to crop growers and advising them to substitute chemical fertilizers with environmentally-friendly alternatives can help organic crops expand under rainfed and supplementary irrigation conditions. Therefore, this research aimed to evaluate a novel aspect: the impact of exogenously applied ALA on the physiological responses, antioxidant activity, and yield of *Lens culinaris*. Lentils are largely used as ingredients in diverse food applications. The study will be conducted under supplemental irrigation and rainfed conditions.

## Materials and methods

### Plant materials and experimental setup

**Figure 1:**
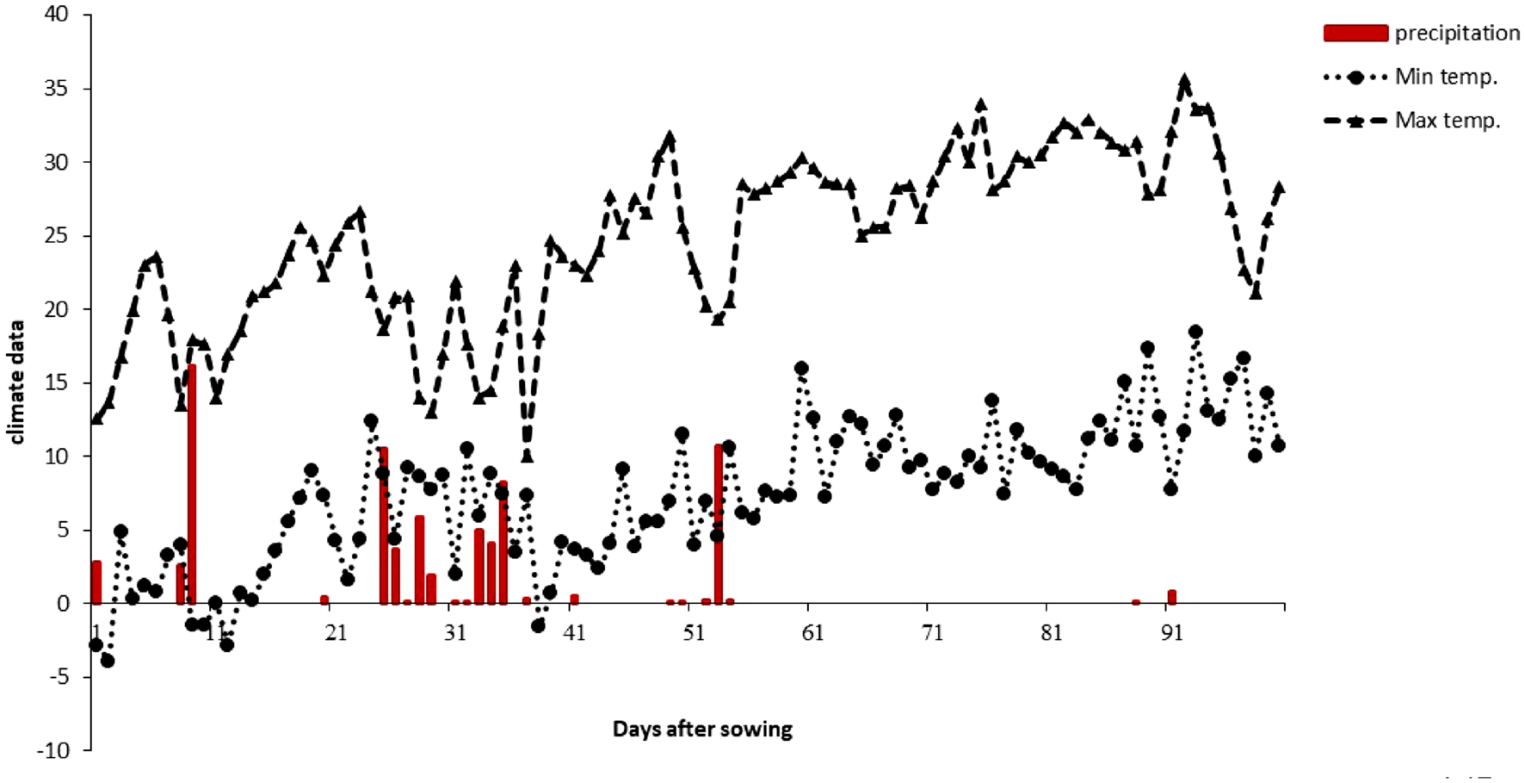
Daily weather data [maximum (Max) and minimum (Min) air temperature (°C) and precipitation (mm)] for the period of cultivation in experimental site.

Field experiments were conducted to investigate the effects of 5-aminolevulinic acid (ALA) on the growth and yield of lentil plants from April to July 2021 in a research farm located in East Azarbaijan province (Varzeghan City), Iran. The climatic data of the region in the period of field trials is presented in Fig. 1. The physical and chemical properties of the soil are presented in Table 1.

**Table 1:** Physio-chemical characteristics of soil used in this study.

Clay (%)	Silt (%)	Sand (%)	pH	Ec (mS/m)	O.C (%)	K (av.) mg/kg	P (av.) mg/kg	T.N.V (%)	T.N (%)
22	38	40	7.99	0.86	0.79	194	8.3	14.5	0.073

The research was carried out as a split plot in the form of a randomized complete block design (RCBD) with three replications (*n* = 3). The main plot included supplementary irrigation treatment at four levels [(supplementary irrigation in the flowering stage, supplementary irrigation in the flowering and early seed filling stages, supplementary irrigation in the early seed filling stage, and rainfed conditions (no irrigation)] and sub-plot factor was foliar application of 5-aminolevulinic acid (ALA) at three levels (0, 50 and 100 ppm). In each replication, there were 5 rows of cultivation with a length of 12 m. The distance between the rows was 15 cm and the distance between the plants on the row was 5 cm. The lentil seeds (Bilehsevar variety) were provided by Agriculture and Natural Resources Research of East Azerbaijan (Iran), and planted on 4 April 2021. The first supplementary irrigation was done in the flowering stage (R2) on June 6, 2021. Simultaneously with irrigation in the evening of the same day, foliar spraying of 50 and 100 ppm ALA was done for supplementary irrigation treatments in the R2 stage for three consecutive days. On 23 June 2021, the pods were formed and at this stage, on 26 June 2021, the second supplementary irrigation (R5) took place. Next, in the evening of the same day, foliar spraying of 50 and 100 ppm ALA was applied for supplementary irrigation treatments in the early seed stage (R5) for three consecutive days, and at the time of harvest (12 July 2021), 10 plants were randomly selected from each plot. By removing the marginal effects, it was selected and immediately transferred to the laboratory to measure the morphological traits. Also, from each plot, one square meter was selected by removing the marginal effects, and at the end of the growth period, it was harvested and considered for the measurement of some other morphological parameters. In addition, from 10 plants, leaf samples were used to measure photosynthetic pigments, cellular injury indices, Malondialdehyde (MDA) and H_2_O_2_ levels, and antioxidant enzymes activity.

### Determination of yield related parameters

In this experiment, the characteristics of plant height, number of pods per plant, number of seeds per pod, weight of 1000 seeds, harvest index (Eq. 1), as well as biological and economic yield, nitrogen content, and seed protein were measured.

$$\mathrm{Harvest \;index }\left(\mathrm{\%}\right)=\frac{{\mathrm{Seed \;yield }(\mathrm{kg}.\mathrm{ha }}^{-1})}{\mathrm{Biological \;yield }{(\mathrm{kg}.\mathrm{ha }}^{-1})} 1$$

#### Plastid pigment measurements

The fresh plant material (0.1 g each) was ground in a mortar with 20 mL of distilled acetone. The extract was centrifuged for 10 min at 8000 × g. The clear supernatant was made up to 10 mL with 80% acetone. The absorbance of the extract was read at 470 (carotenoids), 645 (chlorophyll α), and 663 (chlorophyll b) nm^14^.

#### Determination of H_2_O_2_ content

Fresh tissue material (0.1 g) was extracted with a 0.1% (w/v) trichloroacetic acid (TCA) solution and centrifuged (for 15 min at 12,000×*g*). Then, the supernatant (0.5 mL) was added to 0.5 mL of potassium phosphate buffer (10 mM, pH 7.0) and 1 mL of potassium iodide (1 M). The absorbance of the supernatant was measured at 390 nm. The content of H_2_O_2_ was calculated by comparison with a standard calibration curve previously made by using different concentrations of H_2_O_2_, and H_2_O_2_ concentrations were expressed as μmol g^−1^ fresh weight^15^.

#### Assessment of leaf MDA content

The lipid peroxidation status of leaf samples was analyzed by quantifying their malondialdehyde (MDA) content by the thiobarbituric acid (TBA) reaction. A leaf subsample (100 mg, FW) was homogenized in 5 mL of 0.1% TCA. The solution was centrifuged at 10,000×*g* for 5 min. Then, 1 mL of the supernatant was added to 4 mL of a solution containing 20% TCA and 0.5% TBA. The mixture was heated at 95 °C for 30 min and quickly cooled in an ice bath. Samples were subsequently centrifuged at 12,000×*g* for 10 min. The absorbance of the supernatant was read at 532 nm and the value for the non-specific absorption was deduced at 600 nm^16^. The MDA concentration was determined using its extinction coefficient of 155 mm^−1^ cm^−1^.

#### Estimation of leaf proline content

Free proline was extracted from leaf tissues using aqueous sulphosalicylic acid and quantified using the ninhydrin method^17^. A leaf subsample (0.1 g, FW) was homogenized in a pestle and mortar with 10 mL of 3% aqueous sulphosalicylic acid and briefly centrifuged. 2 mL of glacial acetic acid and ninhydrin were added to 2 mL of supernatant and mixed. This mixture was kept in a boiling water bath for 1 h and the reaction was terminated by placing samples in an ice bath. 4 mL of toluene was added to the mixture, and mixed vigorously for 20–30 s. The chromophore (toluene) layer was aspirated and left to warm up at room temperature. The sample absorbance was recorded at 520 nm, against a reagent blank. The proline content of the samples was determined using a standard curve prepared using different concentrations of pure proline and expressed based on the fresh weight of the samples.

#### Determination of total carbohydrate

The total carbohydrates were extracted from 100 mg of samples with 80% ethanol at 80˚C for 30 min. The process was repeated three times. The supernatants pooled and then dried with a vacuum evaporator (the dried extract was dissolved in distilled water, and diluted to a 100 ml volume to determine the concentrations of total carbohydrates). A calorimetric method was used for determining the concentrations of total carbohydrates^18^. Results were expressed in mg g^−1^ dry matter.

#### Antioxidant enzyme assays

Protein content was determined according to the method of Bradford with BSA as standard. The peroxidase activity (POD) was measured according to the method of Chance and Maehly^19^ at 470 nm using a spectrophotometer. For the measurement, a 3-mL reaction mixture was prepared with the following composition: 50 mM potassium phosphate buffer (pH 7), 20 mM guaiacol, 40 mM H_2_O_2_, and 0.1 mL of enzyme extract obtained from plant leaves. The reaction was initiated by adding the enzyme extract.

To assess the catalase (CAT) activity, 50 µL of enzyme extract, 600 µL of phosphate sodium buffer with a pH of 7, 0.15 µL of EDTA, and 549.85 µL of distilled water were combined in a tube. Subsequently, 382.5 µL of H_2_O_2_ was added to the mixture. To create a solution of 0.75 M H_2_O_2_, 385.5 µL of H_2_O_2_ was mixed with 2.5 mL of distilled water. From this solution, 30 µL was added to the reaction mixture to achieve a concentration of 15 mM H_2_O_2_. The spectrophotometer was employed to measure the absorbance at a wavelength of 240 nm. The absorbance was then recorded again after 60 s, following the methodology outlined by Aebi^20^.

The determination of ascorbate peroxidase (APX) activity in the purified protein was conducted using ascorbate oxidation, as previously described with minor modifications^21^. The enzymatic activity of the total recombinant protein and fractions separated by size exclusion chromatography (SEC) was monitored by measuring the reduction in absorbance at 290 nm. The activity was measured immediately after the addition of 1 mM H_2_O_2_ to a 500 μL reaction mixture containing 0.5 mM ascorbate, 50 mM potassium phosphate buffer (pH 7.0), and AtAPX1 protein, using a UV–visible spectrophotometer for 3 min. Potassium cyanide (KCN 8 mM) was utilized as an inhibitor of heme-containing protein.

The determination of superoxide dismutase (SOD) activity was conducted by adding 50 µl of the enzymatic extract to a solution consisting of 13 mm l-methionine, 75 µm p-nitroblue tetrazolium chloride (NBT), 100 µm EDTA, and 2 µm riboflavin in a 50 mm potassium phosphate buffer with a pH of 7.8. The reaction took place in a chamber under the illumination of a 30 W fluorescent lamp at 25 °C. The reaction was started by turning the fluorescent lamp on and stopped 5 min later by turning it off^22^. The resulting blue formazane, produced through NBT photoreduction, was quantified by measuring the increase in absorbance at 560 nm.

#### Nitrogen concentration and protein content

The last harvest was used to take samples for N determination. 20 g of sample was extracted from each plot. The plant samples were subsequently subjected to oven-drying at a temperature of 75 °C for 48 h, after which they were pulverized using an electric mill. Subsequently, the total nitrogen content was assessed utilizing the Kjeldahl method described by Devani et al.^23^. The percentage of protein content was calculated by multiplying the Kjeldahl nitrogen by the conversion factor of 6.25 (Eq. 2)^24^.

2 $${\text{Seed protein }}\left( \% \right) = {\text{nitrogen content}} \times {6}.{25}$$

### Statistical analysis

All data were analyzed using SAS software version 9.1. The obtained data were expressed as mean ± standard error (*n* = 3). Statistical differences were evaluated by Duncan’s multiple range test (DMRT) using two-way analysis of variance (ANOVA). A significant difference is guaranteed at *p* < 0.05.

### Consent for publication

All authors have agreed to submit the manuscript in its current form for consideration and possible publication in “*Scientific Reports*”.

### Statement on experimental research and field studies on plants

The cultivated plants sampled comply with relevant institutional, national, and international guidelines and domestic legislation of Iran.

### Statement specifying permission

The lentil seeds (Bilehsevar variety) were provided by Agriculture and Natural Resources Research of East Azerbaijan (Iran). For this study, we acquired permission to perform this experiment issued by the Agricultural and Natural Resources Ministry of Iran.

## Results and discussion

### Analysis of variance

The results of the statistical analysis indicate that supplementary irrigation had a significant impact on various aspects of the plant growth, including plant height, number of pods per plant, number of seeds per pod, 1000-seed weight, biological yield, seed yield, harvest index, seed nitrogen, seed protein, chlorophyll a, chlorophyll b, carotenoid, total chlorophyll, total soluble sugar, proline content, MDA content, H_2_O_2_ content, POX activity and CAT activity. Additionally, the foliar application of ALA was found to be significant for a number of parameters, including number of seeds per pod, biological yield, 1000-seed weight, seed nitrogen, seed protein, chlorophyll a, chlorophyll b, carotenoid, total chlorophyll, total soluble sugar, MDA content, H_2_O_2_ content, POX activity and CAT activity. Moreover, there was a significant interaction between the effects of supplementary irrigation and foliar ALA application on plant height, number of seeds per pod, seed yield, total soluble sugar, total chlorophyll, carotenoid, chlorophyll b, chlorophyll a, seed protein, seed nitrogen, CAT activity, POX activity, H_2_O_2_ content and MDA content (Table 2). This two-way interaction indicates that the interpretation of the main effects is incomplete or avoided and it will be discussed in detail below. The discussion will start with the highest-level interactions and move on to the main effects of treatments.

**Table 2:** Analysis of variance (ANOVA) for the studied traits in Lentil plants exposed to exogenous application of ALA under rain-fed and supplemental irrigation conditions.

Source of variation	*df*	Mean square													
		Plant height	No. pods per plant	No. seeds per pod	1000- seed weight	Biological yield	Seed yield	Harvest index	Seed nitrogen	Seed protein	Chlorophyll *a*	Chlorophyll *b*	Carotenoid	Total chlorophyll	Total soluble sugar
Block	2	0.10^ ns^	0.36^ ns^	0.021*	0.002^ ns^	1075.28^ ns^	3941.66^ ns^	0.0013^ ns^	0.00045*	0.016*	0.00014^ ns^	0.0005*	0.0005^ ns^	0.0004^ ns^	0.78^ ns^
Supplemental Irrigation	3	25.55**	36.07**	0.235**	69.705**	31,456.41**	369,933.43**	0.0921**	0.24156**	9.433**	0.02335**	0.0036**	0.0329**	0.0235**	83.82**
Main error	6	0.45	0.77	0.007	0.242	1695.74	4301.40	0.0015	0.00008	0.003	0.00004	0.0006	0.0016	0.0007	0.21
ALA	2	9.84^ ns^	2.11 ^ns^	0.050**	17.037**	3330.86**	2558.96^ ns^	0.0006^ ns^	1.76800**	69.126**	0.00289**	0.0096**	0.0368**	0.0221**	19.14**
Supplemental irrigation × ALA	6	0.31**	1.41^ ns^	0.064**	0.290^ ns^	354.75^ ns^	11,502.11 **	0.0037**	0.01927**	0.754**	0.00010^ ns^	0.0009**	0.0112**	0.0005**	0.77**
Error	15	0.16	0.71	0.006	0.153	623.92	2138.11	0.0007	0.00007	0.003	0.00010	0.0001	0.0015	0.0002	0.28
Source of variation															

Source of variation	*df*	Mean square													
		Proline content	MDA content	H2O2 content	POX activity	CAT activity	APX activity	SOD activity							
Block	2	3.28 ns	0.007 ns	0.015 ns	0.02 ns	20.71 ns	0.26 ns	369.405 ns							
Supplemental irrigation	3	23.04*	3.345**	2.516**	1.94**	39.33*	0.79 ns	423.237 ns							
Main error	6	6.33	0.012	0.011	0.06	5.37	0.72	425.064							
ALA	2	7.52 ns	0.263**	0.067*	1.48**	0.03 ns	2.67**	1951.508**							
supplemental irrigation × ALA	6	3.94 ns	0.066**	0.075**	0.30**	33.60*	0.35 ns	405.299 ns							
Error	15	6.59	0.005	0.015	0.05	9.60	0.34	266.336							

*Significant at the 0.05 probability level; **Significant at the 0.01probability level; ns: Not significant.

According to the results, the highest height (29.83 cm) was observed in the plants treated with 100 ppm of ALA and complementary irrigation during flowering and seed-filling stages. In contrast, the lowest plant height (23.77 cm) belonged to the control group (without ALA application) under rain-fed conditions (Table 3). Previous studies have shown that the external application of ALA can affect the process of net photosynthesis, photosynthetic carbon assimilation, and consequently, increase the height and biomass production in plants. Water scarcity is known to cause leaf wilting, which leads to a reduction in turgor pressure and consequently affects growth processes, particularly longitudinal growth. Photosynthesis is also affected due to the closure of stomata which reduces CO_2_ absorption, ultrastructure of cytoplasmic and enzymatic activity disorder, reduced photosynthetic radiation absorption by canopy, and reduced light use efficiency.

**Table 3:** Effect of exogenous application of ALA on plant height, seed nitrogen and protein contents, photosynthetic pigments (chlorophyll and carotenoid) and total soluble sugar values in lentil plants under different drought stress conditions.

Supplementary irrigation	ALA treatment	Height (cm)	Seed nitrogen (%)	Seed protein (%)	Chlorophyll *b* (mg g^−1^ FW)	Total chlorophyll (mg g^−1^ FW)	Carotenoid (mg g^−1^ FW)	Total soluble sugar (mg g^−1^ DW)
In the flowering and early seed filling stages	0	28.33 ± 0.33	3.72 ± 0.1 g	23.27 ± 0.03 g	0.07 ± 0.01 bc	0.25 ± 0.01 cd	0.36 ± 0.05 ab	17.03 ± 0.04 cd
	50	29.33 ± 0.32	4.20 ± 0.2 d	26.25 ± 0.05 d	0.13 ± 0.02 ab	0.31 ± 0.02 b	0.45 ± 0.03 ab	18.85 ± 0.57 b
	100	29.83 ± 0.17	4.67 ± 0.06 a	29.19 ± 0.04 a	0.17 ± 0.01 ab	0.37 ± 0.03 a	0.45 ± 0.01 ab	20.67 ± 0.33 a
Supplementary irrigation in the flowering stage	0	27.17 ± 0.16	3.70 ± 0.03 g	23.15 ± 0.1 g	0.08 ± 0.01 bc	0.20 ± 0.01 f.	0.35 ± 0.02 ab	12.27 ± 0.13 h
	50	28.83 ± 0.18	3.84 ± 0.04 f.	23.98 ± 0.05 f.	0.09 ± 0.01 bc	0.24 ± 0.02 de	0.37 ± 0.01 ab	13.36 ± 0.16 fg
	100	28.65 ± 0.19	4.37 ± 0.1 c	27.31 ± 0.07 c	0.09 ± 0.01 bc	0.26 ± 0.01 cd	0.48 ± 0.02 a	14.05 ± 0.40 ef
Supplementary irrigation in the early seed filling stage	0	26.37 ± 0.35	3.73 ± 0.1 g	23.28 ± 0.08 g	0.04 ± 0.2 c	0.22 ± 0.02 ef	0.46 ± 0.03 a	14.93 ± 0.37 e
	50	27.70 ± 0.29	3.97 ± 0.11 e	24.81 ± 0.08 e	0.09 ± 0.01 bc	0.27 ± 0.01 c	0.44 ± 0.01 ab	16.80 ± 0.16 d
	100	27.50 ± 0.39	4.43 ± 0.12 b	27.71 ± 0.09 b	0.11 ± 0.03 ab	0.30 ± 0.03 b	0.44 ± 0.01 ab	17.92 ± 0.37 c
Rain-fed conditions	0	23.77 ± 0.34	3.49 ± 0.08 h	21.81 ± 0.05 h	0.08 ± 0.01 bc	0.14 ± 0.01 g	0.17 ± 0.01 b	11.24 ± 0.40 i
	50	25.77 ± 0.15	3.72 ± 0.06 g	23.25 ± 0.1 g	0.12 ± 0.02 ab	0.20 ± 0.02 f.	0.35 ± 0.01 ab	12.56 ± 0.15 gh
	100	26.17 ± 0.17	4.20 ± 0.1 d	26.25 ± 0.03 d	0.13 ± 0.02 ab	0.22 ± 0.02 ef	0.40 ± 0.01 ab	12.85 ± 0.33 gh

*Means followed by the same letter(s) in each column are not significantly different based on Duncan’s Multiple Range Test (n = 3).

The use of complementary irrigation during the flowering stage and early seed-filling phase has been found to increase the height of lentils under rain-fed conditions (Table 3). This helps to reduce the effects of low soil moisture on lentils, providing the water they require during this critical growth stage. As a result, the plant height is increased. The height of the plant is determined by the number of nodes and internode length. Research has shown that the number of plant nodes increases with the availability of nutrients, solubility, and absorption, while moisture can increase the distance between two consecutive nodes^25^. In previous studies, environmental stress conditions have been found to decrease plant height. However, ALA plays a crucial role as a vital precursor in the tetrapyrrole biosynthetic pathway, and is widely recognized as a plant growth regulator that effectively increases plant physiology and fortifies water stress resilience, particularly in rain-fed environments^26^.

Results showed that the highest number of pods per plant was related to supplementary irrigation in the flowering and early grain-filling stages, and the lowest number of pods per plant was due to the dry conditions (Table 4). According to previous studies, the number of pods per plant was positively correlated with grain yield^27^. It has been reported the number of pods per plant is influenced by environmental conditions such as water, high temperature, and planting density. Since environmental stresses occur during the early reproductive period, the yield potential can be adversely impacted, and the number of pods formed in plants usually has the highest reaction to the environmental stresses^27^. The reproductive stage is one of the sensitive phases of plant growth. Under drought stress, fewer pods are formed in the plant, and irrigation after the reproductive period does not have much effect on the number of pods in the plant. Previous studies have shown that drought stress can reduce grain yield by 43%. As the number of pods per plant has a high correlation with grain yield, the number of pods also decreases by 44% under drought stress. This is due to the limitations of the transfer and production of photosynthetic materials during periods of stress^28^. Although supplementary irrigation during flowering and early seed filling can increase the number of pods, the difference in the number of pods between plants receiving supplementary and dry irrigation treatments is likely due to the continued vegetative growth in plants with supplementary irrigation. However, when it comes to complementary irrigation, the role of indeterminate lentil growth should not be overlooked.

**Table 4:** Mean comparison of morpho-physiological traits in lentil plants with or without exogenous application of ALA under different drought stress conditions.

Supplementary irrigation	No. pods per plant	1000 seed weight (g)	Biological yield (Kg ha^−1^)	Chlorophyll *a* (mg g^−1^ FW)	Proline content (µmol g ^−1^ FW)
Irrigation in the flowering and early seed filling stages	11.78 a	54.31 a	1903.37 a	0.19 a	3.03 b
Irrigation in the flowering stage	9.44 b	51.21 c	1889.11 a	0.15 c	4.05 ab
Irrigation in the early seed filling stage	8.00 c	53.45 b	1857.70 a	0.18 b	4.97 ab
Rain-fed conditions	7.22 c	48.07 d	1771.48 b	0.08 d	6.78 a

ALA foliar application	1000-seed weight (g)	Biological yield (Kg ha^−1^)	Chlorophyll *a* (mg g^−1^ FW)	APX activity (µmol H_2_O_2_ decomposited min^−1^ mg^−1^ protein)	SOD activity (U mg^−1^ protein)
Control	50.74 c	1836.78 b	0.14 c	2.04 b	33.89 b
50 ppm	51.47 b	1860.61 a	0.15 b	2.90 a	56.44 a
100 ppm	53.07 a	1868.86 a	0.17 a	2.80 a	55.50 a

*Means followed by the same letter(s) in each column are not significantly different based on Duncan’s Multiple Range Test (n = 3).

**Figure 2:**
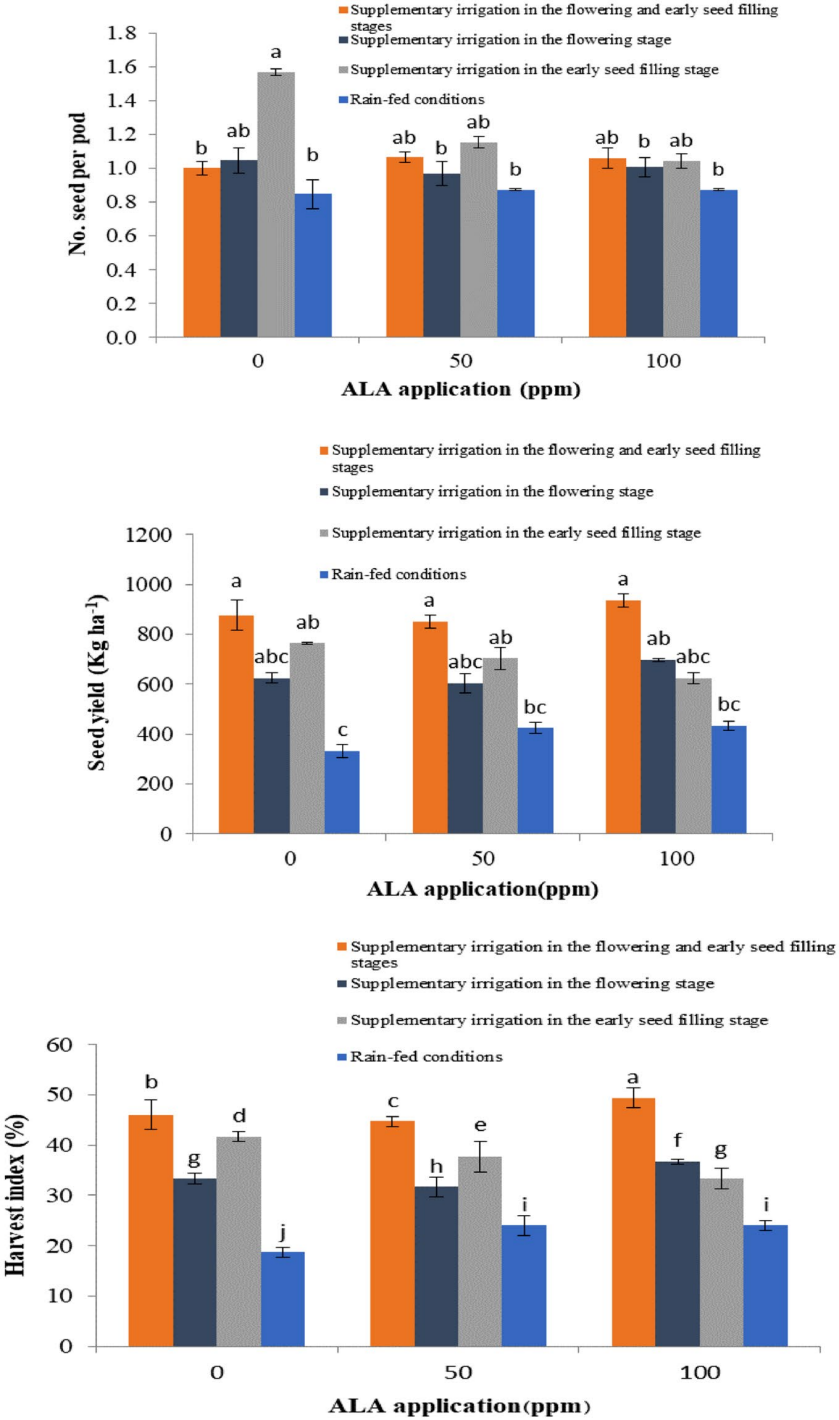
Mean comparison of yield related traits in lentil plants treated with exogenous application of 5-aminolevulinic acid (ALA) under different drought stress conditions. Columns with different letters show significant differences at *P* ˂ 0.01 using Duncan’s multiple range test.

The study found that the plants treated with foliar ALA application at 50 ppm and the control group without ALA, both under supplementary irrigation during the flowering phase, produced the highest number of seeds per pod. Lentil plants treated with 100 ppm ALA under supplementary irrigation in the flowering and early seed filling stages also showed a high number of seeds per pod. However, exogenous application of ALA resulted in the lowest number of seeds in pods during all three stages under rain-fed conditions (Fig. 2).

The number of seeds in pods is a crucial component for the yield in legumes and is mainly affected by genetics. Research shows that the number of seeds per pod is less sensitive to stress conditions due to genetic control, which minimizes the impact of environmental factors. However, recent studies reveal that the number of seeds per pod is the least in rain-fed conditions^29,30^. Our study indicates that the use of exogenous ALA can mitigate the harmful effects of low-water availability stress on lentil plants. Further, it results in a significant increase in the number of seeds per pod, as shown in Fig. 2. Such findings align with previous research that reports the efficacy of ALA in mitigating the adverse consequences of various environmental stresses. ALA protects chlorophyll and the photosynthetic apparatus, stimulates the defensive response, and promotes plant growth^29^. The study shows that lentil plants treated with ALA under complementary irrigation in flowering and seed-filling stages had the highest 1000-seed weight, while the lowest was observed in non-treated plants under rain-fed conditions. It should be noted that the seed weight index is affected by genotypic factors, contributing approximately 20–30% of the overall variation. However, environmental conditions during the maturation stage can also affect this index. Irrigation during flowering and seed-filling stages increases the rate of photosynthesis and the duration of the reproductive stage, resulting in a direct effect on seed weight. Moisture limitation during flowering and seed-filling stages reduces the transfer of photosynthetic materials, leading to wrinkled seeds^30^. Drought stress during the reproductive phase affects seed growth and development, ultimately leading to reduced seed weight.

The results of the study indicate that applying ALA to plants during the flowering and seed-filling stages, along with supplemental irrigation, resulted in the highest biological yield (BY) compared to control plants without ALA application under rain-fed conditions. BY was found to be negatively affected by drought stress, which can limit plant growth by altering metabolic activity, cell turgor, hormonal balance, nutrient absorption and accumulation, water relationships, and antioxidant systems. The decrease in BY is attributed to the decreased accumulation of dry matter, which typically occurs during the flowering and pod formation stages. Therefore, supplementary irrigation during these stages can improve BY. This finding has also been confirmed in other plant species such as beans. Conversely, the decrease in available water during the late vegetative and flowering stages of the lentil plant shortens the period of pod formation, leading to fewer branches and yields. However, some studies have indicated that exogenous ALA can increase potassium accumulation, maintain a high K/Na ratio in the roots, and help plants tolerate stress, leading to improved plant growth and nutrient status^31^.

The study found that applying ALA to lentil plants during early seed filling under supplementary irrigation conditions resulted in the highest seed yield. In contrast, the seed yield had the lowest value in rain-fed conditions. Soil moisture content reduction due to dry conditions affected the seed yield significantly by decreasing the size and number of seeds. Seed filling is a complex process that involves various metabolic processes, and it is sensitive to drought and dehydration due to the involvement of different enzymes and transporters in leaves and seeds. Understanding the target locations that adjust the events of seed filling and how they are affected by abiotic stresses is crucial to increase seed quality. Therefore, it is important to know the physio-biochemical and genetic mechanisms that control seed-filling processes under stressful conditions to develop strategies for enhancing stress tolerance^32^. The study showed that supplementary irrigation during the flowering and early seed-filling stages and exogenous application of ALA at 100 ppm resulted in increased lentil yield under rain-fed conditions. Supplementary irrigation, especially in the flowering and early seed-filling stages, improved the seed yield in lentil plants by increasing the number of pods and 1000-seed weight. ALA application led to increased leaf area duration, enhanced photosynthesis activities, and improved seed size by prolonging their filling period and ultimately enhancing the lentil's yield. Our results indicated that different doses of ALA protected lentil plants from the adverse effects of water availability, which significantly increased the yield. The ALA-mediated increase in stress tolerance is related to lower cellular levels of reactive oxygen species (H_2_O_2_) and lower degradation of photosynthetic pigments, resulting in an efficient oxidative defense system.

According to the results, the highest harvest index value of 49.33% was observed in plants treated with ALA, and supplementary irrigation during the flowering stage and early grain filling. On the other hand, the lowest harvest index value of 18.67% was found in plants that were not treated with ALA and were grown under rain-fed conditions. However, when ALA was applied to plants growing under rain-fed conditions, it led to a significant increase in their harvest index. The harvest index is a measure of the success of a genotype in transferring essential absorbed substances in the economic part of the crop, and it can be used as a selection criterion for higher yield^33^. The reason for the increase in the harvest index under supplementary irrigation conditions could be attributed to the 1000-seed weight, number of seeds per pod, and number of pods per plant. This is consistent with previous studies that have shown that drought stress can reduce the harvest index due to a decrease in biological yield and grain yield at different ratios. In addition, the decrease in harvest index is probably due to the impact on performance and further biological performance over grain yield. Similarly, a significant decrease in performance components and harvest index was observed in different wheat genotypes under various levels of drought stress and normal conditions. In another study on various genotypes of lentils, supplementary irrigation, especially in the flowering stage, led to an increase in the harvest index value^34^.

The study found that the highest level of chlorophyll a was observed in plants grown under supplementary irrigation and treated with 100 ppm ALA. However, the lowest level of chlorophyll a was obtained under rain-fed conditions without ALA application. The effect of spraying with ALA on chlorophyll b content was significant at the 1% probability level. The highest chlorophyll b content was obtained under supplementary irrigation in the flowering stage and spraying of 100 ppm ALA, while the lowest chlorophyll b was observed under supplementary irrigation in the early seed-filling stage without foliar ALA application. The highest total chlorophyll content was observed in supplementary irrigation at flowering and early seed-filling stages and exogenous application of 100 ppm ALA, while the lowest total chlorophyll was obtained in rain-fed conditions and without ALA treatment. Drought stress affects various plant processes, including chlorophyll content and stability, which are vital processes. Chlorophyll is an essential photosynthetic pigment in plants that harvests light and generates energy in the form of reducing power. These parameters are commonly used to describe drought resistance in many screening studies^35^. Other studies showed that drought stress in the vegetative stage reduces chlorophyll a, chlorophyll b, and total chlorophyll content in the vegetative and flowering stages. Severe drought stress also inhibits photosynthesis by changing the chlorophyll content, which affects chlorophyll components and damages the photosynthesis apparatus^36^. Leaf chlorophyll content decreases due to drought stress, as reported by Ommen et al^37^. One of the most important criteria for stress tolerance in plants is the chlorophyll stability index^38^. Among the ways to reduce damage to chlorophylls in plants, we can mention optimal osmotic regulation and maintaining RWC. The use of these mechanisms prevents the over-production of ROS and further damage to chlorophyll^38^. ALA, as a key precursor of chlorophyll biosynthesis, has a reinforced role under different environmental stresses. For example, ALA application increased chlorophyll content in lettuce seedlings under exposure to UV-B stress^39^. In another study, ALA application adjusted the chlorophyll fluorescence indicators in rapeseed under drought stress^40^. The role of ALA on oxidative stress and photosynthetic capacity of "Williams" under drought stress in semi-arid conditions was investigated. The exogenous ALA application at concentrations of 15, 30, and 45 mg L^−1^ significantly increased the plant's photosynthetic activity. Chlorophyll reduction in dehydration conditions is mainly due to damage to chloroplasts caused by ROS. However, exogenous ALA can reduce the accumulation of ROS and maintain the stability of the chloroplast membrane under stress environments. In addition, ALA inhibits chlorophyll synthesis by reducing the level of Mg-chelatase and Protochlorophyllide oxidoreductase^41^. The higher concentration (5–40 mM) of ALA led to the accumulation of several chlorophyll intermediaries in plants, such as protochlorophyllide, protoporphyrin IX, and protoporphyrin magnesium^42^. Chlorophyll intermediaries act as a hydroxyl and singlet oxygen formation that causes oxidative damage to the treated plants. ALA shows a variety of regulatory roles and physiological effects such as improving the plant growth index, chlorophyll synthesis, and photosynthesis in plants. However, overusing ALA leads to oxidative damage in plants. The proper concentration of ALA is responsible for satisfactory plant growth. Additionally, the reinforcement effects of ALA on plants may be caused by light and nutrients^29^.

The highest amount of carotenoids was observed in plants that were given supplementary irrigation and 100 ppm ALA application. Conversely, the lowest amount of carotenoids was found in plants grown under rain-fed conditions and without ALA application, as shown in Table 3. Carotenoids are plant metabolites that are derived from the isoprenoid pathway and are soluble in lipids. They are accumulated in various plant organs and play a crucial role in enhancing drought stress tolerance. Carotenoids scavenge ROS and free radicals and help keep a balance of redox^43^. During stressful conditions, plants regulate the turnover of carotenoids by controlling genes that are associated with carotenoid metabolism. Studies have shown that water stress reduces the amount of carotenoids in plants^44^. ALA application enhances carotenoid biosynthesis by positively regulating enzymes such as geranylgeranyl diphosphate synthase, phytoene synthase, phytoene desaturase, and lycopene β-cyclase^41^. In addition, ALA application has been found to increase the content of carotenoids, as well as improve chlorophyll a, chlorophyll b, and total chlorophyll, as demonstrated in another study^45^.

The nitrogen content in lentil seeds decreased significantly under rain-fed conditions (Table 3). When foliar ALA was applied during different phenological stages under varying water availability conditions, it resulted in significant changes in the lentil plant's response to ALA. The application of ALA increased N content in seeds under supplementary irrigation compared to rain-fed conditions. The highest seed protein content was observed in lentil plants that received supplementary irrigation and ALA application (at 100 ppm). As per the results in Table 3, there was a significant increase in the percentage of seed protein under two times of supplementary irrigation compared to one supplementary irrigation and rain-fed conditions. The highest seed protein of lentil plants was obtained by 29.19% under supplementary irrigation during flowering and early seed-filling stages. Additionally, ALA foliar application led to a significant increase in the percentage of lentil seed protein in all irrigation levels. The decrease in soil moisture content disrupts nitrogen fixation by increasing the resistance to oxygen diffusion of root bacteria. This leads to a decrease in nitrogenase activity and ultimately reduces the availability of nitrogen for protein biosynthesis, which is the primary storage in legumes^46^. As a result, seed yield is reduced. However, our study shows that foliar ALA application was the main factor in increasing the percentage of seed protein under both supplementary irrigation and rain-fed conditions.

The quality of seed protein mostly depends on the genotype of the plant, but environmental stresses can also affect it. Drought stress, for instance, can cause changes in protein composition by affecting the total nitrogen content accumulated during seed filling. Studies have shown that under drought stress, beans have reduced levels of iron, zinc, phosphorus, nitrogen, and total protein content^47^. Similarly, white, red, and chitty varieties of beans have shown a significant decrease in nitrogen and seed protein contents under drought stress. Studies on wheat have also shown that drought stress during the seed-filling stage can significantly affect the quality of seed protein. While globulin and glutenin remained unaffected, the concentration of albumin and gliadin increased significantly under drought stress conditions^47^. However, some studies have reported increased seed protein concentration in response to drought stress in various agricultural plant species. It is important to note that these differences might depend on the severity and duration of drought stress.

Results showed that the highest content of MDA and H_2_O_2_ were related to rain-fed conditions and the lowest was obtained in complementary irrigation in flowering and early seed-filling stages with foliar ALA application (Table 5). In other words, complementary irrigation and ALA application resulted in reduced MDA and H_2_O_2_ content. Studies have shown that rapeseed seedlings under water treatment with ALA at low concentrations (1 mg/l) significantly improved plant biomass and chlorophyll contents but reduced MDA and ROS production^49^. Our study also indicated a decline in H_2_O_2_ and MDA contents due to efficient antioxidant systems in plants treated with exogenous ALA.

**Table 5:** Effect of exogenous application of ALA on malondialdehyde (MDA) and hydrogen peroxide (H_2_O_2_) contents, as well as peroxidase (POX) and catalase (CAT) activities in lentil plants under different drought stress conditions.

Drought stress	ALA treatment	MDA content (nmol g^−1^ FW)	H_2_O_2_ content (µmol g^−1^ FW)	POX activity (µmol H_2_O_2_ decomposited min^−1^ mg^−1^ protein)	CAT activity (µmol H_2_O_2_ decomposited min^−1^ mg^−1^ protein)
Supplementary irrigation in the flowering and early seed filling stages	0	0.22 ± 0.01 ef	0.24 ± 0.02 c	0.80 ± 0.19 bc	20.63 ± 1.68 ab
	50	0.08 ± 0.01 g	0.26 ± 0.01 c	2.05 ± 0.04 a	23.29 ± 1.33 a
	100	0.05 ± 0.01 g	0.25 ± 0.01 c	2.29 ± 0.11 a	20.23 ± 1.94 ab
Supplementary irrigation in the flowering stage	0	0.26 ± 0.03 ef	0.26 ± 0.03 c	0.50 ± 0.05 c	23.46 ± 1.67 a
	50	0.22 ± 0.02 ef	0.25 ± 0.01 c	0.98 ± 0.12 b	16.03 ± 1.10 b
	100	0.15 ± 0.02 fg	0.22 ± 0.02 c	0.98 ± 0.21 b	23.29 ± 2.63 a
Supplementary irrigation in the early seed filling stage	0	0.40 ± 0.05 d	0.27 ± 0.03 c	0.69 ± 0.03 bc	16.24 ± 0.43 b
	50	0.33 ± 0.04 de	0.40 ± 0.09 c	0.74 ± 0.21 bc	22.89 ± 2.15 a
	100	0.23 ± 0.02 ef	0.27 ± 0.02 c	0.98 ± 0.16 b	19.30 ± 1.49 ab
Rain-fed conditions	0	1.82 ± 0.13 a	1.67 ± 0.02 a	0.48 ± 0.05 c	18.29 ± 2.36 ab
	50	1.36 ± 0.08 b	1.19 ± 0.06 b	0.89 ± 0.04 bc	16.09 ± 0.36 b
	100	1.09 ± 0.05 c	1.11 ± 0.07 b	0.83 ± 0.14 bc	15.86 ± 2.43 b

*Means followed by the same letter(s) in each column are not significantly different based on Duncan’s Multiple Range Test (n = 3).

**Figure 3:**
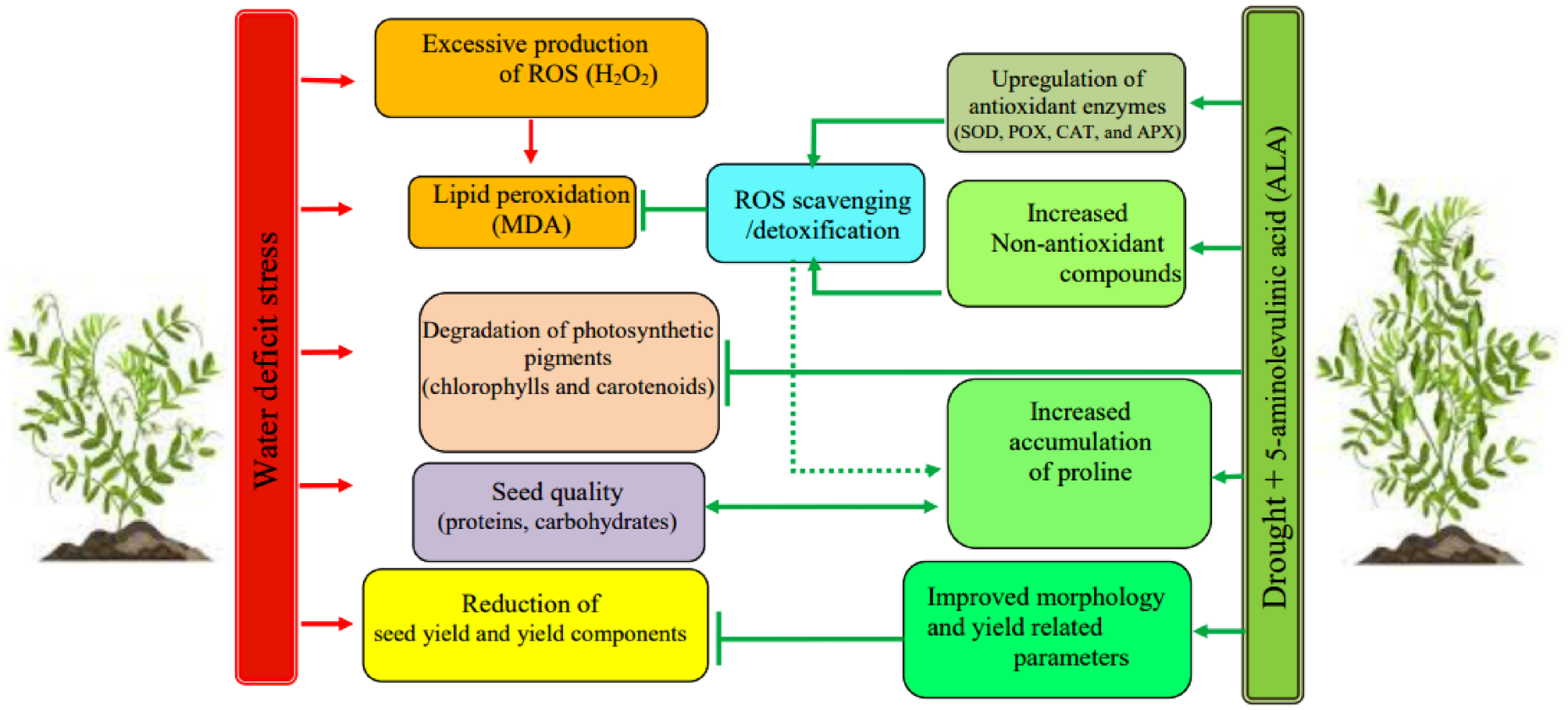
Proposed mechanism for the 5-aminolevulinic acid (ALA)-induced mitigation of drought negative effects in lentil plants.

Results showed that the highest proline content was obtained under water stress, especially in dry-farming conditions (Table 4). Plants can accumulate compatible solutes, such as proline, as an adaptive mechanism to endure abiotic stress. In our study, we observed a substantial increase in cellular proline levels under rainfed conditions. The addition of ALA resulted in a significant increase in proline levels. Proline also acts as an antioxidant by effectively scavenging toxic reactive oxygen species (ROS). A similar study on the enhancement of proline content through ALA was discovered in *Brassica napus*^50^ subjected to salt stress and in sunflower under water deficit stress^29^. The exogenous application of ALA increased proline contents, which may have led to a reduction in ALA biosynthesis, while simultaneously promoting proline biosynthesis. Further research is required to determine whether the influence of exogenous application of ALA on proline accumulation is correlated with a decline in ALA biosynthesis^48^. According to the results, ALA application increased total soluble carbohydrate contents at all stress levels, but this rate was higher in complementary irrigation conditions during flowering and early seed-filling stages (Table 3). The quantity and quality of compounds in plant organs, especially pulses, change in different moisture conditions. Osmotic stress in plants is a frequently observed consequence of water deficit conditions. Nevertheless, carbohydrates also fulfill additional crucial roles within the plant^52^. For instance, carbohydrates play a pivotal role in regulating redox homeostasis, gene expression, enzyme activities, stomatal opening, and ion transport in plants when subjected to abiotic stressors. Furthermore, a noteworthy increase in the accumulation of overall carbohydrates in lentils was observed under precipitation-dependent circumstances. The exogenous administration of ALA led to a further escalation in this parameter. Drought stress induces oxidative damage in plants due to incomplete destruction of ROS. However, the detoxification of ROS highly relies on better oxidative defense in the form of enzymatic and non-enzymatic compounds. The activities of antioxidant enzymes, including SOD, POD, and CAT were investigated to establish the roles of ALA in response to osmotic stress in lentils. Supplementary irrigation in flowering and early seed filling stages with foliar ALA application led to a significant increase in POX and CAT enzyme activity in lentil plants under rain-fed conditions. The maximum activity in supplementary irrigation conditions in flowering stage with foliar ALA application at 100 ppm was obtained and the lowest POX and CAT enzymes activity was obtained under rain-fed conditions and without ALA application (Tables 4, 5). Studies show that ALA plays an important role in the plant's response to adverse environmental conditions. ALA can significantly reduce oxidative damage caused by drought through increasing antioxidant enzyme activity such as SOD, CAT, and POD and preservation of chloroplast structure. ALA can also improve abscisic acid (ABA) levels by increasing nutrient absorption, auxin levels, and cytokinin^49^. An increase in antioxidant enzyme activity has been reported in different plant species in response to ALA application^50^. In other studies, ALA showed a significant effect in increasing antioxidant enzyme activity, antioxidative compounds, and photosynthetic pigments and was suggested as a compound that mitigates stress and improves seed quantity and quality^51^. A schematic model for the ALA-induced mitigation of drought-negative effects in lentil plants is given in Fig. 3. Therefore, the activation of key antioxidant enzyme activities by ALA may play a pivotal role in mitigating osmotic stress in lentils. Our study has additionally revealed a decrease in H_2_O_2_ and MDA concentrations due to the efficient antioxidant mechanism in plants that have undergone exogenous ALA treatment.

## Conclusions

The study revealed the positive effects of using exogenous application of ALA on lentils growth and metabolism under rainfed conditions. By providing the supplementary irrigation during flowering and seed-filling stages, the quality and growth of lentils can be increased, resulting in improved yield-related traits. Moreover, foliar application of ALA enhances the studied attributes in lentil when grown under rain-fed conditions. Exogenous ALA regulates the photosynthetic pigments (chlorophylls and carotenoids), antioxidant enzyme activities, and compatible solutes while reducing H_2_O_2_ levels and increasing yield components in lentil plants, making them more tolerant to water-deficit stress. The use of ALA as a growth regulator is a novel approach to mitigate drought stress effects in dry farming conditions, and further research is needed to evaluate the regulatory mechanism of ALA treatment at the molecular level.

## Data Availability

All the data generated/ analyzed during the study are available with the corresponding author on reasonable request.
